# Effectiveness of Dietary Allergen Exclusion Therapy on Eosinophilic Colitis in Chinese Infants and Young Children ≤ 3 Years of Age

**DOI:** 10.3390/nu7031817

**Published:** 2015-03-11

**Authors:** Min Yang, Lanlan Geng, Peiyu Chen, Fenghua Wang, Zhaohui Xu, Cuiping Liang, Huiwen Li, Tiefu Fang, Craig A. Friesen, Sitang Gong, Dingyou Li

**Affiliations:** 1 Department of Gastroenterology, Guangzhou Women and Children’s Medical Center of Guangzhou Medical University, Guangzhou 510623, China; E-Mails: 273459720@qq.com (M.Y.); genglan_2001@hotmail.com (L.G.); chenpei.y@163.com (P.C.); xuer_hui@126.com (Z.X.); 172262276@qq.com (C.L.); Lookfory@hotmail.com (H.L.); fangtf@126.com (T.F.); 2 Department of Pathology, Guangzhou Women and Children’s Medical Center of Guangzhou Medical University, Guangzhou 510623, China; E-Mail: wangfhua@163.com; 3 Division of Gastroenterology, Children’s Mercy Hospitals and Clinics, Kansas City 64108, MO, USA; E-Mail: cfriesen@cmh.edu

**Keywords:** eosinophilic colitis, infants, children, dietary exclusion

## Abstract

Eosinophilic colitis is a well recognized clinical entity mainly associated with food allergies. Empiric treatment options include dietary allergen exclusion (extensively hydrolyzed protein formula and elimination diet), anti-allergy medications (antihistamines and leukotriene receptor antagonists) and corticosteroids. We evaluated the effectiveness of dietary antigen exclusion on clinical remission of eosinophilic colitis in infants and young children. We retrospectively reviewed charts of all infants and children ≤3 years of age who were diagnosed with eosinophilic colitis (defined as mucosal eosinophilia ≥20 hpf^−1^) from 1 January 2011 to 31 December 2013 at a tertiary children’s hospital in China. Forty-nine children were identified with eosinophilic colitis. Elemental formula, simple elimination diet or combination therapy resulted in clinical improvement in 75%, 88.2% and 80% of patients, respectively. In conclusion, eosinophilic colitis in infants and children ≤3 years of age responded well to dietary allergen exclusion.

## 1. Introduction

Eosinophilic gastrointestinal disorders (EGIDs) are a spectrum of chronic diseases characterized by a range of symptoms and eosinophilic infiltration of the gastrointestinal tract and have been increasingly reported in recent years [1,2,3]. Depending on the segment of gastrointestinal tract involved, various clinical presentations have been described, including eosinophilic esophagitis, eosinophilic gastroenteritis and eosinophilic colitis. Although the underlying pathophysiology of EGIDs is still poorly understood, they have been shown to be strongly associated with food allergies [4,5,6,7]. Thus dietary exclusion of allergenic foods is the key component of a treatment regimen [8,9,10,11].

Among the entities of EGIDs, eosinophilic esophagitis has been well-studied and elemental diet has been shown to be effective in inducing histological remission in more than 90% of children [12,13]. However, the efficacy of elemental diet or dietary elimination in infants and children with eosinophilic colitis has been rarely reported. We hypothesize that elemental diet or dietary exclusion of allergenic foods is an effective treatment in infants and young children with eosinophilic colitis. The aim of this study is to evaluate the effectiveness of dietary antigen exclusion on clinical remission of eosinophilic colitis in Chinese infants and young children ≤ 3 years of age.

## 2. Experimental Section

### 2.1. Study Design and Data Collection

The institutional ethics review committee approved this study protocol.

We retrospectively reviewed charts of all infants and children ≤3 years of age who underwent colonoscopy from 1 January 2011 to 31 December 2013 at Guangzhou Women and Children’s Medical Center (Guangzhou, China). The main indications for colonoscopy were unexplained persistent diarrhea, blood in the stool, abdominal pain/irritability or poor weight gain. We excluded patients with polyps and inflammatory bowel disease. Patients had routine stool culture and parasite examination prior to colonoscopy and those with bacterial infection and parasite infestation were also excluded.

Clinical features, feeding patterns during the first six months of life, laboratory tests and colonic mucosal eosinophil density were analyzed. At least three tissue biopsy samples were obtained from terminal ileum, cecum, ascending, transverse, descending or rectosigmoid colon. Not all patients had biopsies taken from all six sites. The tissue specimens were fixed in buffered 10% formalin, routinely processed, embedded in paraffin, sectioned at 4 μm, stained with hematoxylin and eosin (HE), and examined using light microscopy. At least three paraffin blocks were investigated. The colonic pathological changes were observed and evaluated by an experienced pathologist to determine eosinophil density per high power field (hpf).

### 2.2. Definition of Eosinophilic Colitis

Eosinophilic colitis was defined as mucosal eosinophilia ≥20 hpf^−1^ in one or more colonic biopsy sites [14,15,16]. Those with mucosal eosinophilia <20 hpf^−1^ in all colonic biopsy sites were considered to be non-eosinophilic colitis subjects and served as controls.

### 2.3. Serum Allergen-Specific Immunoglobulin E (sIgE) Test

Total serum IgE and sIgE to milk, egg, fish, shellfish, peanut, soybean, wheat, rice, beef, chicken, lamb, mushroom, tomato and onion were measured using commercially available assays (Specific IgE REAST, Dr. Fooke-Achterrath Laboratorien GmbH, Germany) following the manufacturer’s instructions. The sIgE levels >0.35 IU mL^−1^ were classified as positive.

### 2.4. Treatment Regimen for Eosinophilic Colitis

Eosinophilic colitis patients were treated based on the presence of sIgE. All infants and young children with positive sIgE were treated with dietary allergen exclusion (elemental formula for infants who were on formula feeding or dietary elimination of milk, egg, peanut and wheat for those who were not on formula feeding). For those with negative sIgE, combination therapy with dietary elimination and anti-allergy medications (loratidine, montelukast or ketotifen) was initiated. For those patients who did not have any clinical improvement with dietary exclusion or combination therapy for three months, corticosteroids (oral prednisone or budesonide) were used for three months.

Infants and children with colonic mucosal eosinophilia <20 hpf^−1^ (non-eosinophilic colitis) were not treated according to any standard protocol. Some patients continued follow-up in our clinic and the others went to other facilities for medical advice.

### 2.5. Statistical Analysis

Results are expressed as mean ± SD. Differences between means were tested for statistical significance using a one-way analysis of variance (ANOVA) and post-hoc least significance tests. Differences between proportions were analyzed with the chi-squared test. A *p*-value of less than 0.05 was considered significantly different.

Based upon published studies and our preliminary data, we predicted that the remission rate with dietary antigen exclusion would be 80% in eosinophilic colitis patients and 50% in non-eosinophilic colitis patients. Based on a statistical power of 0.9 to detect a significance (*p* < 0.05, one-sided), 25 patients were required for the eosinophilic colitis group, for which we had 49 patients. Due to the fact that some of the non-eosinophilic colitis patients did not follow up in our medical facility, clinical data was incomplete to compare the efficacy of treatment between the two groups. Instead, we only described the remission rates of the eosinophilic colitis group.

## 3. Results

### 3.1. Patient Selection, Presenting Symptoms and Patient Characteristics

**Figure 1:**
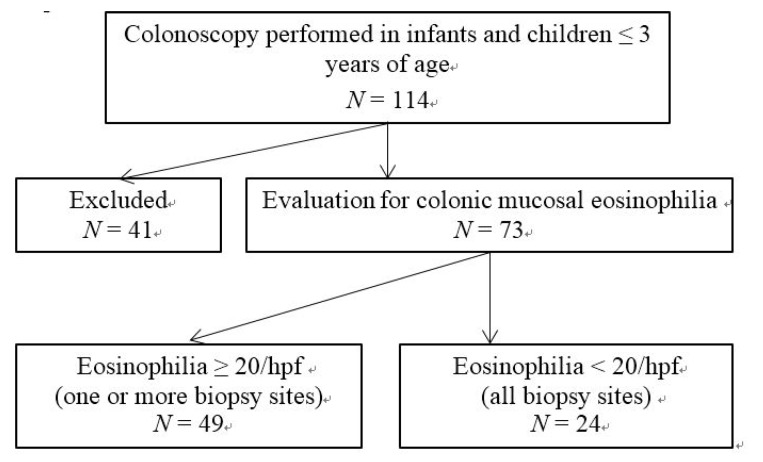
Flow diagram of patient group selection.

A total of 114 infants and young children underwent colonoscopy. As shown in Figure 1, 41 patients (36.0%) were excluded due to colonic polyps and infections. Forty-nine children (43.0%) had colonic mucosal eosinophilia ≥20 hpf^−1^ in one or more colonic biopsy sites and 24 patients (21.0%) had mucosal eosinophilia <20 hpf^−1^.

The distribution of eosinophilic infiltration ≥20 hpf^−1^ varied among different colonoscopy biopsy sites, ranging from 55.6% of biopsy tissues in rectosigmoid colon to 86.1% of biopsy tissues in ascending colon. The mean eosinophilic infiltration among different sites was similar (from 32.1 ± 11.8 hpf^−1^ in transverse colon to 38.2 ± 18.7 hpf^−1^ in ascending colon).

Presenting symptoms for colonoscopy were persistent diarrhea (47.9%), blood in the stool (34.2%), abdominal pain/irritability (11.0%), poor weight gain (4.1%) and paleness (2.7%). As shown in Table 1, patients with eosinophilic colitis were more likely to have blood in the stool than those non-eosinophilic colitis patients, but the difference did not reach statistical significance.

**Table 1:** Presenting symptoms of patients undergoing colonoscopy. Eosinophilic colitis group: colonic mucosal eosinophilia ≥20 hpf^−1^. Non-eosinophilic colitis group: colonic mucosal eosinophilia <20 hpf^−1^.

Symptoms	Eosinophilic Colitis (*n* = 49)	Non-Eosinophilic Colitis (*n* = 24)
Diarrhea	23 (46.9%)	12 (50%)
Blood in stool	20 (40.8%)	5 (20.8%)
Abdominal pain/irritability	4 (8.2%)	4 (16.7%)
Poor weight gain	1 (2.0%)	2 (8.3%
Paleness	1 (2.0%)	1 (8.3%)

Patient’s characteristics, including mean age, sex, feeding patterns and time to add solid foods during the first six months of life, did not differ between the two groups (Table 2).

**Table 2:** Patient basic characteristics in infants and children with or without eosinophilic colitis.

Basic characteristics	Eosinophilic Colitis (*n* = 49)	Non-Eosinophilic Colitis (*n* = 24)
Age (mean ± SD)	20.9 ± 8.8	17.6 ± 8.6
<12 months	12 (24.5.0%)	4 (16.7%)
12 months–36 months	37 (75.5.0%)	20 (83.3%)
Youngest age	3 months	4 months
Sex, Male/Female	33/16	16/8
Feeding patterns:		
Breast feeding	24 (49.0%)	13 (54.2%)
Formula feeding	8(16.3%)	4 (16.7%)
Mixed feeding	17 (34.7%)	7 (29.1% )
Time to add Solid food:		
≤4 months	7 (14.3%)	2 (8.3%)
4–6 months	38 (77.6%)	20 (83.3%)
≥6 months	4 (8.2%)	2 (8.3% )

### 3.2. Clinical Features and Laboratory Tests in Children with Eosinophilic Colitis

As shown in Table 3, no significant differences were found in symptoms between the two patient groups (*p* > 0.05). However, a positive serum allergen-specific IgE test (sIgE) occurred significantly more frequently in children with eosinophilic colitis. Milk and/or egg IgE account for most of the offending allergens. Peripheral blood eosinophil percentage and total serum IgE elevation had a trend to occur more in eosinophilic colitis than non-eosinophilic colitis group but did not reach statistical significance.

**Table 3:** Clinical features and laboratory tests in infants and children with or without eosinophilic colitis. * *p* < 0.05.

Symptoms and Laboratory Tests	Eosinophilic Colitis (*n* = 49)	Non-Eosinophilic Colitis (*n* = 24)
Abdominal pain	11 (22.4%)	6 (25.0%)
Nausea/vomiting	13 (26.5%)	7 (29.1%)
Blood in stool	32 (65.3%)	10 (41.7%)
Poor appetite	10 (20.4% )	4(16.7%)
Bloating/distention	6 (12.2% )	3 (12.5%)
Irritability with stooling	7 (14.3%)	3 (12.5%)
Bristol Stool Form Scale 1–2	8 (16.3%)	2 (8.3%)
Bristol Stool Form Scale 6–7	19 (38.8%)	9 (37.5%)
Stool OB (+)	22 (44.9%)	8 (33.3%)
Anemia (HB < 110 g L^−1^)	15(30.6%)	8 (33.3%)
Blood eosinophil > 5%	21 (42.9%)	6(25.0%)
Total serum IgE elevation	22 (44.9%)	7 (29.2%)
Hypoalbuminemia (<30 g L^−1^)	4 (8.2%)	1 (4.2%)
sIgE (+)	29 (59.2%) *	7(29.1%)
milk	22 (44.9%)	6 (12.5%)
egg	14 (28.5%)	3 (12.5%)
Milk + egg	11(22.4%)	2 (8.3%)

### 3.3. Treatment Outcomes for Infants and Children with Eosinophilic Colitis

**Figure 2:**
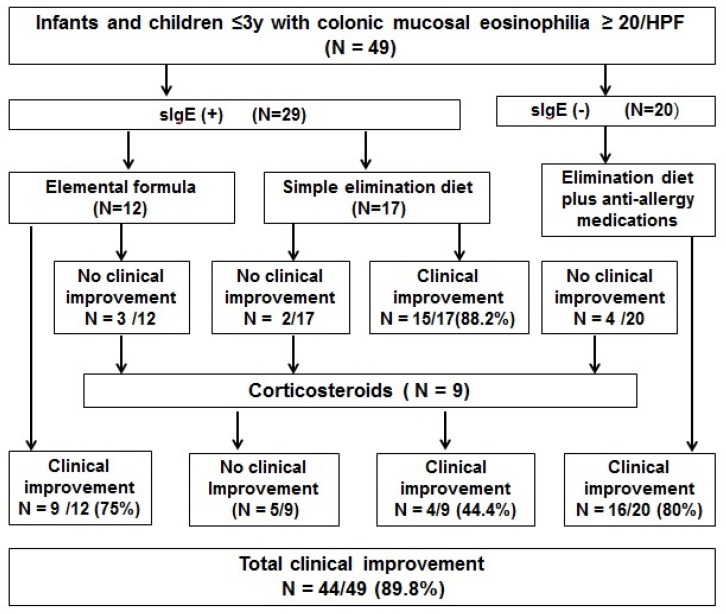
Treatment outcomes for infants and children with eosinophilic colitis.

The treatment outcomes of all patients with eosinophilic colitis are shown in Figure 2. Elemental formula, simple elimination diet or combination therapy resulted in clinical improvement in 75%, 88.2% and 80% of patients, respectively, at three months’ clinic follow-up. More specifically, 24 (82.3%) of the 29 sIgE-positive patients and 16 (80%) of the 20 sIgE-negative patients responded to initial treatment with dietary antigen exclusion. For those nine patients who did not respond to those therapies, four patients (44.4%) responded to a three-month therapy with corticosteroids (prednisone, *n* = 8; budesonide, *n* = 1). Altogether, 44 of the 49 (89.8%) patients with eosinophilic colitis had clinical improvement.

Of note, infants and children with colonic mucosal eosinophilia <20 hpf^−1^ were not treated according to any standard protocol. Some patients continued follow-up in our clinic and the others went to other facilities for medical advice. Fifteen of the 24 infants and children were followed up in our facility and their clinical charts were reviewed. Five patients had a positive sIgE and were treated with dietary elimination (*n* = 2), elemental formula (*n* = 1) or anti-allergy medication (*n* = 2). The remaining 10 patients received probiotics (*n* = 4) or no specific treatment (*n* = 6). Altogether, seven of the 15 patients (46.7%) had clinical improvement and three worsened. Two patients underwent repeat colonoscopy and were subsequently diagnosed with eosinophilic colitis (mucosal eosinophilia ≥ 20 hpf^−1^) and responded to oral corticosteroids.

## 4. Discussion

We performed a retrospective chart review of infants and young children with eosinophilic colitis and their treatment outcomes. Our results showed that eosinophilic colitis defined as colonic mucosal eosinophilia ≥20 hpf^−1^ was common in infants and children ≤3 years of age undergoing colonoscopy. Furthermore, infants and children with mucosal eosinophilia ≥20 hpf^−1^ responded well to dietary allergen exclusion or combination therapy of dietary elimination and anti-allergy medications. To our knowledge, this is the first study to investigate colonic mucosal eosinophilia in this defined patient group and to examine its clinical relevance in treatment.

There is still a lack of agreement on the definition of normal colonic eosinophil density in infants and children. Lowichik and Weinberg (1996) examined the intestinal tract mucosa from 44 infants and children who died suddenly and unexpectedly and found a mean colonic eosinophil count of 17 eosinophils hpf^−1^ [17]. DeBrosse *et al.* (2006) examined histologically normal gastrointestinal biopsies of children and observed a mean eosinophil density of 20.3, 16.3 and 8.3 eosinophils hpf^−1^ in cecum, transverse colon and rectum, respectively [18]. Behjati *et al.* (2009) showed that the mean colonic eosinophil count in children with a diagnosis of colonic eosinophilia was 16.4 hpf^−1^ [14]. Saad (2011) established a normal number of eosinophils in the cecum, ascending, transverse, descending and rectosigmoid colon as 14.2 ± 6.1, 12.0 ± 6.1, 11.9 ± 4.6, 10.7 ± 5.6 and 12.4 ± 6.1 hpf^−1^, respectively [19]. Although there is still no consensus, the presence of colonic mucosal eosinophilic infiltration ≥20 hpf^−1^ is generally considered indicative of eosinophilic colitis [14,15,16].

Eosinophilic colitis is a well recognized clinical entity mainly associated with food allergies. However, its prevalence, diagnostic criteria and treatment standards have not been clearly defined. Lozinsky & Morails (2014) systemically reviewed the literature for clinical data on infants with eosinophilic colitis and found that eosinophilic infiltration (between 5 and 25 hpf^−1^) in colonic or rectal biopsy was seen in 89.3% (236/264) of infants younger than 24 months [16]. However, the authors did not report the prevalence of eosinophilic infiltration ≥20 hpf^−1^ in their data analysis. In our study, we found that colonic mucosal eosinophilia ≥20 hpf^−1^ occurred in 43% of infants and young children ≤3 years of age who underwent colonoscopy for unexplained persistent diarrhea, blood in the stool, abdominal pain/irritability or poor weight gain. Our study would be the first reported prevalence of eosinophilic colitis in this specific patient population.

We found no association between colonic eosinophil density and clinical features in terms of gastrointestinal symptoms, demographics, feeding patterns and laboratory tests with the exception of sIgE test. Our findings are consistent with the report by Behjati *et al.* (2009), who showed no significant difference in patients’ symptoms and other characteristics among children with mean colonic eosinophil levels at <10 hpf^−1^, 10–20 hpf^−1^ and ≥20 hpf^−1^ [14]. Therefore, clinical features and routine laboratory tests are not sufficient to differentiate infants and young children with or without eosinophilic colitis.

There have been no prospective randomized controlled trials on any specific therapy for eosinophilic colitis. Empiric treatment options include dietary allergen exclusion (extensively hydrolyzed protein formula and elimination diet), anti-allergy medications (antihistamines and leukotriene receptor antagonists) and corticosteroids [15,16,20,21]. Since cow’s milk protein allergy is the main cause of eosinophilic colitis in infants and children, a formula containing extensively hydrolyzed protein or amino acids is recommended as initial therapy [22]. In our study, we found that a positive slgE test was significantly more frequent in children with eosinophilic colitis and milk and/or egg accounted for most of the offending allergens, further supporting initial dietary exclusion therapy. Friesen *et al.* (2004 & 2006) showed that children with gastrointestinal mucosal eosinophilia responded well to a leukotriene receptor antagonist, combined H1/H2 antagonists and cromolyn [23,24]. Ketotifen, a second-generation H1-antagonist, has been shown to be safe and effective in treating eosinophilic gastroenteritis [25]. Chen *et al.* (2003) treated 13 patients with a diagnosis of eosinophilic gastroenteritis with oral prednisolone and the symptoms in all the patients subsided within two weeks [26]. There have been no controlled studies of corticosteroids on eosinophilic colitis. In our clinical practice, infants and children with eosinophilic colitis were initially treated with dietary exclusion (elemental formula or elimination diet) if the slgE test was positive or with combination therapy of an elimination diet plus anti-allergy medication if the slgE test was negative. We demonstrated that 75%–88.2% of infants and children with mucosal eosinophilia ≥20 hpf^−1^ responded well to dietary allergen exclusion or combination therapy. With our treatment regimen, 44 of the 49 (89.8%) patients with eosinophilic colitis had clinical improvement.

EGIDs are recognized to result from immunoglobulin E (IgE)-mediated immediate, mixed or non-IgE-mediated chronic reactions. In our study, 59.2% of eosinophilic colitis patients had positive sIgE tests, which is consistent with reports by others [7,27,28]. Positive sIgE to cow’s milk protein was found in 44% of infants suspected of milk protein allergy [27]. Erwin *et al.* (2010) showed that sIgE measurements identified previously undiagnosed food sensitivity in 42% of children with eosinophilic esophagitis [28]. However, the relationship between sIgE and pathogenic mechanism of eosinophilic colitis remains unclear and needs to be explored in a large controlled study. Interestingly, eosinophilic colitis patients with positive or negative sIgE tests seemed to respond to dietary antigen exclusion therapy in a similar way (82.8% *vs.* 80%), although those with negative sIgE tests were also treated with anti-allergic medications at the same time. We speculate that eosinophilic colitis in those patients with negative sIgE tests was caused by non-IgE-mediated allergic reactions. Our study implies that empiric dietary antigen exclusion therapy was effective in infants and young children with eosinophilic colitis regardless of sIgE status. Rodriguez-Sanchez *et al.* (2014) recently reported a similar result in patients with eosinophilic esophagitis and found that both sIgE-targeted elimination diets and empiric six-food elimination diets had similar histological remission rate (73% *vs.* 53%, *p* = 0.17) [29].

The major limitation of this study is that this study was a retrospective analysis of treatment outcome with no placebo control. We were able to identify 15 of the 24 infants and children with colonic eosinophilia <20 hpf^−1^ and only 46.7% had clinical improvement after trials of various treatments. Even though this was not a randomized controlled study, our results strongly suggest that infants and children with eosinophilic colitis responded better to dietary exclusion than those without eosinophilic colitis.

Another limitation is the lack of consensus for the histological definition of eosinophilic colitis and variability of normal mucosal eosinophil density in different segments of colon. It is reasonable to argue that eosinophilia ≥20 hpf^−1^ in one segment (e.g., rectosigmoid) indicates eosinophilic colitis while in another segment (e.g., cecum) may be normal. However, there has been no agreement on which biopsy site to be used to determine colonic eosinophilia. Since repeat colonoscopy after treatment is not clinically indicated, we did not have data available to analyze the histological remission rate. A well-designed multicenter controlled study with pre- and post colonoscopy with biopsies taken from each site in all patients would be needed to determine histological remission and whether mucosal eosinophilia in a specific segment would be better to define eosinophilic colitis.

## 5. Conclusions

Our retrospective study demonstrated that eosinophilic colitis was common in infants and children ≤3 years of age who underwent colonoscopy in a tertiary children’s medical center in southern China. Overall, those patients responded well to our treatment regimen consisting of initial dietary allergen exclusion and/or anti-allergy medications and subsequent corticosteroid therapy. Further prospective trials are necessary to confirm our findings.
